# 异基因造血干细胞移植急性移植物抗宿主病诊断与治疗中国专家共识（2024年版）

**DOI:** 10.3760/cma.j.cn121090-20240608-00214

**Published:** 2024-06

**Authors:** 中华医学会血液学分会干细胞应用学组

**Keywords:** 异基因造血干细胞移植, 急性移植物抗宿主病, Allogeneic hematopoietic stem cell transplantation, Acute graft-versus-host disease

## Abstract

尽管异基因造血干细胞移植（allo-HSCT）的疗效不断改善，急性移植物抗宿主病（GVHD）仍然是主要的合并症和死亡原因。近年来，随着急性GVHD防治新型药物的出现及系列临床研究的更新，常规急性GVHD预防及治疗方案有着不同程度的变化。基于近年来在该领域的主要研究成果和临床经验的积累，本共识在《中国异基因造血干细胞移植治疗血液系统疾病专家共识——急性移植物抗宿主病（2020年）》基础上做进一步更新。

## Abstract (en)

Despite the continuous improvement in the efficacy of allogeneic hematopoietic stem cell transplantation（allo-HSCT）, acute graft-versus-host disease（GVHD）remains a major complication and cause of death. In recent years, with the emergence of new drugs for the prevention and treatment of acute GVHD and the update of a series of clinical studies, there have been varying degrees of changes in the routine prevention and treatment regimens for acute GVHD. Based on the main research achievements and the accumulation of clinical experience in this field in recent years, this consensus further updates the “The Consensus on Allogeneic Hematopoietic Stem Cell Transplantation for Hematological Diseases in China-Acute Graft-Versus-Host Disease（2020）.

异基因造血干细胞移植（allo-HSCT）是治疗多种血液系统疾病的有效方法。尽管allo-HSCT的疗效不断改善，急性移植物抗宿主病（GVHD）仍然是主要的合并症和死亡原因。近年来，随着急性GVHD防治新型药物的出现及系列临床研究的更新，常规急性GVHD预防及治疗方案有着不同程度的变化。基于近年来在该领域的主要研究成果和临床经验的积累，本共识在《中国异基因造血干细胞移植治疗血液系统疾病专家共识——急性移植物抗宿主病（2020年）》基础上作进一步更新。

一、急性GVHD的定义和发生率

（一）急性GVHD的定义

GVHD指由异基因供者细胞与受者组织发生反应导致的临床综合征。美国国立卫生研究院（NIH）的定义将急性GVHD分为经典急性GVHD和晚发急性GVHD：经典急性GVHD一般指发生在移植后100 d（+100 d）以内，且主要表现为皮肤、胃肠道和肝脏三个器官的炎性反应；晚发急性GVHD指具备经典急性GVHD的临床表现、但发生于+100 d后的GVHD。晚发急性GVHD包括以下几种情况：+100 d后新发生的急性GVHD、已缓解的经典急性GVHD在+100 d后再激活、经典急性GVHD延续至+100 d后。当急性GVHD表现和慢性GVHD同时存在时，诊断为重叠慢性GVHD。供者淋巴细胞输注（DLI）后急性GVHD诊断以DLI时间为计时起点，其他与移植后急性GVHD诊断标准相同[1]–[2]。

（二）急性GVHD的发生率

国内资料显示，异基因造血干细胞移植后中度和重度急性GVHD发生率为13％～47％，发生率的差异主要与危险因素有关[3]–[16]。在同胞全相合移植中，Ⅱ～Ⅳ、Ⅲ/Ⅳ度急性GVHD的发生率分别为13％～35％、5.0％～7.7％[3]–[7]；在非血缘供者移植中，Ⅱ～Ⅳ、Ⅲ/Ⅳ度急性GVHD的发生率分别为12.5％～47.0％、6.6％～13.5％[8]–[10]；在单倍体移植中，Ⅱ～Ⅳ、Ⅲ/Ⅳ度急性GVHD的发生率分别为18.5％～43.9％、7.9％～13.8％[11]–[14]；在脐血干细胞移植中，Ⅱ～Ⅳ、Ⅲ/Ⅳ度急性GVHD发生率分别为28.0％～30.6％、15.0％～19.4％[15]–[16]。

二、急性GVHD的危险因素

在中国，单倍体移植主要采用基于粒细胞集落刺激因子（G-CSF）和抗胸腺细胞球蛋白（ATG）的非体外去T细胞移植模式（北京方案）。单倍体移植中急性GVHD的危险因素与同胞全相合移植和非血缘供者移植并不完全相同，而且通过改进急性GVHD的预防方案可不同程度地削弱这些危险因素的作用[6],[17]。

（一）移植类型

既往认为急性GVHD与HLA不合的程度有关。近年来大量资料表明，在同胞全相合移植、非血缘供者移植和单倍体移植三种移植类型中，重度急性GVHD的发生率并无明显差异[3]–[5]。采取优化供者选择及基于危险度的GVHD分层预防措施后，Ⅱ～Ⅳ度急性GVHD的发生率在单倍体移植和非血缘供者移植中呈降低趋势[6],[11]–[12],[17]。

（二）供患者HLA不相合的位点数量

在非血缘供者移植中，急性GVHD发生率随着HLA不相合位点数量增加而增高[4],[9]。在单倍体移植中，Ⅱ～Ⅳ度急性GVHD和HLA不相合位点数量无关[13],[17]。

（三）性别与年龄

在同胞全相合移植中，供者为女性（尤其是多次妊娠者）的患者具有较高的急性GVHD发生率，男性患者与女性供者、老年患者与老年供者均为GVHD的危险因素[6],[18]。而在单倍体移植中，母亲或非遗传性父体抗原（NIPA）不合同胞供者为急性GVHD的危险因素[17]。

（四）移植物来源及成分

针对移植物来源，在同胞全相合和非血缘供者移植中发现应用外周血干细胞可能增加急性GVHD发生风险[19]–[20]。单倍体移植北京方案模式下G-CSF动员的移植物中，与骨髓血联合外周血干细胞相比，单纯外周血干细胞采集物并不增加急性GVHD发生率[21]。单倍体移植中移植物组分也影响急性GVHD的发生和分度，包括CD4/CD8比值、CD3/CD14比值、CD8阳性细胞绝对值等[22]–[23]。

（五）急性GVHD预防方案

急性GVHD预防方案与急性GVHD的发生密切相关，针对高危患者进行急性GVHD预防方案的改进可减弱危险因素的作用，如在环孢素A（CsA）+短程甲氨蝶呤（MTX）方案基础上增加霉酚酸酯（MMF）等能够降低急性GVHD的发生率、减轻严重程度[24]。同胞全相合或非血缘供者造血干细胞移植中，ATG或CD52单抗的应用可降低急性GVHD的发生率[19]。单倍体移植北京方案模式下，母系或旁系供者移植后加入低剂量环磷酰胺可有效降低急性GVHD的发生率[12]。

三、急性GVHD的药物预防

（一）同胞相合移植

1. CsA联合短程MTX[25]：①CsA：起始剂量1.5 mg/kg每12 h 1次静脉滴注，−1 d开始（也有主张−7 d或−9 d开始），有效谷浓度150～250 µg/L，消化道症状消失后改为口服。一般情况下恶性疾病移植后3个月CsA渐减，6个月停用，但应根据复发风险和GVHD情况酌情缩短或延长CsA应用时间；良性疾病（如重型再生障碍性贫血）移植后1年CsA减停，根据嵌合体和GVHD情况酌情缩短或延长CsA应用时间。当CsA不耐受时可更换为他克莫司，初始剂量0.02～0.03 mg·kg^−1^·d^−1^持续输注，一般有效血浓度为7～12 µg/L，消化道症状消失后改为口服给药，减量原则同CsA。②MTX：+1 d 15 mg/m²，+3 d、+6 d 10 mg/m²，静脉滴注给药。回顾性研究结果提示在同胞全相合移植中+11 d是否应用MTX对急性GVHD没有影响[25]–[26]。每次MTX用药结束24 h后采用甲酰四氢叶酸钙解救。

2. MMF及其他：在上述基础预防方案基础上可加用MMF。MMF用法：成人或体重>35 kg儿童1.0 g/d，小儿一般30 mg·kg^−1^·d^−1^，分2～3次口服。MMF一般和CsA同时开始应用（或+1 d开始给药），植活后停用[27]。40岁以上人群加用低剂量兔抗人胸腺细胞球蛋白（rATG）可进一步降低GVHD发生率[28]。

（二）单倍体移植和非血缘供者移植

1. CsA+短程MTX+MMF+ATG：CsA用法及用量同上。恶性血液系统疾病移植100 d后CsA渐减，移植后6～9个月停用（可根据复发风险和急性GVHD情况进行调整）；重型再生障碍性贫血等良性疾病移植后1年减停CsA。如CsA不耐受，可改为他克莫司，用法同上。

MTX用法同上，在重度口腔黏膜炎时+11 d MTX可不用。

MMF用量和开始时间同上，在非血缘供者移植和单倍体移植，植活后停用[27],[29]。

ATG在国内最多应用的是rATG，推荐总剂量7.5～10.0 mg/kg，−5 d～−2 d分次输注[14],[30]。

2. 其他：针对GVHD高危患者开展的一些探索，如在单倍体移植中以生物标志（骨髓移植物的CD4^+^细胞/CD8^+^细胞比值）为指导分层短期应用低剂量糖皮质激素、在母系或旁系单倍体移植后加用低剂量环磷酰胺，均能有效降低急性GVHD发生率[12],[28]。

（三）脐血干细胞移植

1. CsA联合MMF：CsA和MMF用法与同胞全相合移植相同。CsA也可采用持续静脉滴注方式给药，平均血药浓度200～300 µg/L。如恶性血液病无GVHD迹象，一般移植后2个月CsA开始逐渐减量，至少用至移植后6个月。

2. 关于ATG的应用：既往脐带血干细胞移植多用ATG，近年来有学者认为不用ATG也是可行的。孙自敏等[31]采用清髓性预处理方案联合CsA+MMF预防GVHD进行非血缘脐血干细胞移植，对照组为清髓性预处理方案联合CsA+MMF+MTX或ATG-Fresenius S（7.5 mg·kg^−1^·d^−1^×3 d）预防GVHD进行的非血缘脐血干细胞移植，两组间Ⅱ～Ⅳ、Ⅲ/Ⅴ度急性GVHD的发生率差异均无统计学意义，而不含ATG组的植入率、无病生存率和总生存率明显优于使用ATG组，差异有统计学意义。

（四）DLI[32]–[33]

DLI一般输注G-CSF动员的淋巴细胞，也可输注直接采集的供者淋巴细胞，其主要风险是GVHD发生率和致死率增高，不同类型DLI的GVHD预防方案有所不同。

1. 预防性DLI：一般情况下，在给予预防性DLI时CsA尚在应用中，DLI时将CsA调整至有效浓度。DLI后CsA的应用时间因移植类型（同胞全相合移植或单倍体移植）、DLI距移植的时间、输注细胞种类（G-CSF动员与否）而有所差异。

2. 干预性DLI：CsA在DLI前1 d开始应用并维持有效血药浓度。持续时间依不同移植类型而定，建议同胞全相合移植患者DLI后4～6周减停，单倍体移植患者DLI后6～8周减停。干预性DLI后也可单用MTX预防GVHD，DLI后1、4、8 d各给药1次，以后10 mg每周1次，共4～6次。

3. 治疗性DLI：一般采用CsA，用法与干预性DLI相同。黄晓军等认为治疗性DLI后短期应用免疫抑制剂可预防重症GVHD发生，而未影响移植物抗白血病（GVL）效应；在单倍体移植后血液学复发的患者中，DLI后采用MTX预防急性GVHD比用CsA可更好保留GVL效应。MTX用法同干预性DLI。也有人认为既往无重症GVHD病史的患者，同胞全相合移植的治疗性DLI后用可MTX或不用药物预防。

（五）其他急性GVHD预防方案

1. 雷帕霉素靶蛋白（mTOR）抑制剂：西罗莫司在急性GVHD预防中的疗效已被证实。在同胞全相合和非血缘供者移植中，在钙调蛋白抑制剂、MMF或MTX基础上加用西罗莫司的三药预防方案均使急性GVHD发生率明显下降[34]–[36]。在同胞全相合、非血缘供者和单倍体移植中，用mTOR抑制剂替代钙调蛋白抑制剂的GVHD预防方案也在不断探索[37]–[38]。

2. 移植后大剂量环磷酰胺（PT-Cy）在同胞全相合、非血缘及单倍体移植后中预防急性GVHD的疗效均被证实[39]。

3. 其他：阿巴西普（Abatacept）是一种选择性T细胞共刺激信号阻断剂，被FDA批准用于GVHD预防方案。在HLA不合非血缘供者移植中，与钙调磷酸酶抑制剂+MTX相比，加用阿巴西普的三药联合可明显降低Ⅲ/Ⅴ度急性GVHD的发生率（2.3％）；钙调磷酸酶抑制剂+MTX联合阿巴西普组与钙调磷酸酶抑制剂+MTX联合ATG组急性GVHD发生率相似[40]。此外，CD52单克隆抗体阿仑单抗（alemtuzumab）、α4β7GI整合素受体拮抗剂维多珠单抗（Vedolizumab）在急性GVHD中预防的研究也在更新，疗效尚需更高级别证据支撑[39]。

四、急性GVHD的鉴别诊断和严重程度分度标准

急性GVHD的诊断和分度主要依赖皮肤、胃肠道和肝脏的受累情况。

（一）急性GVHD临床表现[41]–[42]

1. 皮肤：皮肤是急性GVHD最多累及的靶器官，表现为斑丘疹，多始于头颈部、耳后、面部和肩部，多累及手掌、足心。患者常无明显不适或仅有轻度瘙痒、疼痛。

2. 胃肠道：胃肠道是急性GVHD第二位受累的靶器官，上消化道和下消化道均可累及。上消化道急性GVHD主要表现厌食消瘦、恶心呕吐，下消化道急性GVHD表现为水样腹泻、腹痛、便血和肠梗阻。下消化道急性GVHD与移植后非复发相关死亡密切相关。

3. 肝脏：肝脏急性GVHD表现为胆汁淤积导致的高胆红素血症、伴有或不伴有肝脏酶谱增高、DLI后急性GVHD患者仅表现肝脏酶谱增高，一般认为属于慢性GVHD，也有学者认为表现更似急性GVHD。

4. 其他表现：随着单倍体移植的广泛开展，除了上述三大器官典型急性GVHD表现之外，临床医师观察到疑似免疫原因导致发热和肺、中枢神经系统损伤的现象，有学者认为可能是急性GVHD的特殊表现，因为临床上鉴别诊断非常困难，这些表现是否归于急性GVHD尚有待进一步研究。

（二）诊断及鉴别

急性GVHD主要为临床诊断，需要注意排除其他可能情况，尤其在急性GVHD表现不典型或治疗效果欠佳时，鉴别诊断尤为重要。

皮肤急性GVHD需要与导致皮疹发生的其他情况（预处理毒性、药疹或感染性皮疹等）进行鉴别；重度急性GVHD可扩展至全身，表现为大疱甚至表皮剥脱，与Stevens-Johnson综合征或中毒性表皮坏死松解症进行鉴别。鉴别困难时可考虑皮肤活检[39]。

当患者食欲不振、恶心和呕吐时，可能为上消化道急性GVHD，仅表现上消化道症状时需要和念珠菌病、疱疹病毒感染和非特异性胃炎相鉴别。上消化道急性GVHD的诊断：食欲不振伴体重下降、恶心持续至少3 d，或每天至少2次呕吐持续至少2 d。确诊需要胃或十二指肠活检病理结果。

当腹泻作为急性GVHD初始表现时，应注意与引起腹泻的其他原因相鉴别，包括感染（艰难梭菌、巨细胞病毒、EB病毒、腺病毒、轮状病毒等）、药物不良反应、预处理毒性、血栓性微血管病、消化性溃疡等。血清生物标志物（如TNFR1、TIM3、ST2、REG3α或Elafin等组合）、肠道菌群动态变化可应用于胃肠道急性GVHD的鉴别诊断及预后判断[43]–[45]。

当诊断肝脏急性GVHD时需与引起高胆红素血症的其他疾病相鉴别，如预处理相关毒性、药物性肝损伤、肝窦阻塞综合征、脓毒症相关胆汁淤积和病毒性肝炎等。肝活检诊断急性GVHD应在权衡风险和获益后谨慎采用。

（三）急性GVHD的分度标准

急性GVHD的严重程度分度标准是根据急性GVHD对移植后非复发相关死亡的影响程度制定的，采用皮肤、胃肠道和肝脏急性GVHD分别积分后再形成总的分度。主要有三种分度标准，其中临床最常采用改良Glucksberg标准（表1），近年来急性GVHD国际联盟（MAGIC）分级标准应用有增多趋势（表2）[1],[46]，此外还有IBMTR分级系统[47]–[48]。

**表1:** 改良的急性移植物抗宿主病（GVHD）Glucksberg分级标准

项目	累及器官		
	皮肤	肝脏-胆红素血症	胃肠道
分级			
1级	皮疹面积<25%^a^	总胆红素2.0~3.0 mg/dl^b^	腹泻量>500 ml/d ^c^或持续性恶心^d^
2级	皮疹面积25%~50%	总胆红素3.1~6.0 mg/dl	腹泻量>1 000 ml/d
3级	皮疹面积>50%，全身红斑	总胆红素6.1~15.0 mg/dl	腹泻量>1 500 ml/d
4级	全身红皮病伴大疱形成	总胆红素>15.0 mg/dl	严重腹痛和（或）肠梗阻
分度^e^			
Ⅰ度	1~2级		
Ⅱ度	1~3级	1级	1级
Ⅲ度		2~3级	2~4级
Ⅳ度^f^	4级	4级	

**注** ^a^使用9分法或烧伤图表确定皮疹程度；^b^以总胆红素表示范围（如果已经记录了导致总胆红素升高的其他原因，则将其降一级）；^c^腹泻量适用于成人，≤14岁儿童患者腹泻量应基于体表面积计算（如果记录了腹泻的另一个原因，则将其降一级）；^d^持续恶心并有胃/十二指肠GVHD的组织学证据；^e^作为授予该等级所需的最低器官受累程度的分级标准；^f^Ⅳ度也可能包括较少的器官受累，但功能状态极度下降

**表2:** 急性移植物抗宿主病（GVHD）国际联盟（MAGIC）分级标准

分级	皮疹（仅活动性红斑）	肝脏	上消化道	下消化道（排便）
0级	无活动性（红斑）GVHD皮疹	总胆红素< 2.0 mg/dl	无或间歇性恶心、呕吐或厌食	成人：<500 ml/d或<3次/d 儿童：<10.0 ml·kg^−1^·d^−1^或<4次/d
1级	<25%	总胆红素2.0~3.0 mg/dl	持续性恶心、呕吐或厌食	成人：500~999 ml/d或3~4次/d 儿童：10.0~19.9 ml·kg^−1^·d^−1^或4~6次/d
2级	25%~50%	总胆红素3.1~6.0 mg/dl		成人：1 000~1 500 ml/d或5~7次/d 儿童：20.0~30.0 ml·kg^−1^·d^−1^或7~10次/d
3级	>50%	总胆红素6.1~15.0 mg/dl		成人：>1 500 ml/d或>7次/d 儿童：>30.0 ml·kg^−1^·d^−1^或>10次/d
4级	全身红斑（> 50%）伴水疱形成或表皮剥脱（>5%）	总胆红素>15.0 mg/dl		严重腹痛伴或不伴肠梗阻或便血（无论排便量如何）

**注** 整体临床分级（基于最严重的靶器官受累）：0度：无任何器官1～4级；Ⅰ度：1～2级皮肤，无肝脏、上消化道或下消化道受累；Ⅱ度：3级皮疹和（或）1级肝脏和（或）1级上消化道和（或）1级下消化道；Ⅲ度：2～3级肝脏和（或）2～3级下消化道，0～3级皮肤和（或）0～1级上消化道；Ⅳ度：4级皮肤、肝脏或下消化道受累，0～1级上消化道受累。儿童：≤14岁

五、疗效评估标准及糖皮质激素耐药急性GVHD的定义

急性GVHD开始治疗后每天评估疗效，及时识别糖皮质激素无效的患者。

（一）疗效评估标准

疗效评估通过各个靶器官的急性GVHD分级和整体分度与初始急性GVHD情况的比较获得。完全缓解（CR）指所有受累器官的急性GVHD表现完全消失；部分缓解（PR）指所有初始受累器官的急性GVHD改善（至少降低一个级别）但未达到CR，无其他任何靶器官急性GVHD恶化；无反应（NR）指任何器官的急性GVHD严重程度无改善也没有恶化或患者死亡；进展（PD）指至少1个靶器官的急性GVHD加重（至少增加1个级别）、伴或不伴其他器官急性GVHD的改善。PD和NR为治疗无效[1],[25]。

（二）糖皮质激素耐药急性GVHD的定义

《Thomas' Hematopoietic Cell Transplantation Stem Cell transplantation》（第5版）将一线治疗3 d评估为PD、7 d评估为NR或14 d未达CR的情况定义为糖皮质激素耐药。在2018年欧洲骨髓移植学会-NIH-国际骨髓移植研究中心（EBMT-NIH-CIBMTR）的标准命名中，急性GVHD疗效评估时，将一线糖皮质激素开始治疗后3～5 d内疗效评估为PD或治疗5～7 d内疗效评估为NR或包括糖皮质激素在内的免疫抑制剂治疗28 d未达CR定义为糖皮质激素耐药。此外，将一线治疗糖皮质激素不能减量或减量过程中急性GVHD再激活定义为糖皮质激素依赖。糖皮质激素耐药和糖皮质激素依赖统称为糖皮质激素治疗失败[1]。

六、急性GVHD的治疗

原则上Ⅰ度急性GVHD可密切观察和局部治疗，Ⅱ度及以上急性GVHD诊断后应立即开始一线治疗，但在非血缘供者移植和单倍体移植中早期发生的急性GVHD往往进展较快，也应立即开始一线治疗。

（一）一线治疗

1. 糖皮质激素：最常用甲泼尼龙，推荐起始剂量1 mg·kg^−1^·d^−1^或2 mg·kg^−1^·d^−1^（分2次静脉注射），同时将CsA谷浓度调整至150～250 µg/L并及时评估糖皮质激素疗效[25]。若疗效评估为有效，急性GVHD达CR后缓慢减少糖皮质激素用量，成年患者一般每5～7 d减量甲泼尼龙10～20 mg/d（或等效剂量其他类型糖皮质激素），4周减至初始量的10％。儿童患者参照成人按比例缓慢减量。若判断为糖皮质激素耐药，需加用二线药物，并减停糖皮质激素；如判断为糖皮质激素依赖，二线药物起效后减停糖皮质激素。

2. 糖皮质激素联合用药一线治疗急性GVHD

（1）糖皮质激素联合MTX：黄晓军团队应用低剂量MTX（静脉10 mg或口服15 mg）联合低剂量甲泼尼龙（0.5 mg·kg^−1^·d^−1^）一线治疗急性GVHD，总有效率达81％，皮肤、胃肠道和肝脏急性GVHD的有效率分别为88％、75％、81％[49]。一项Ⅱ期前瞻性研究证实微剂量（5 mg/m^2^）MTX联合标准剂量甲泼尼龙（1 mg·kg^−1^·d^−1^）一线治疗急性GVHD，Ⅰ、Ⅱ、Ⅲ度急性GVHD的完全缓解率分别为100％、82％、66％[50]。另一项多中心Ⅲ期前瞻性随机对照研究表明，微小剂量MTX联合糖皮质激素一线治疗急性GVHD的疗效优于糖皮质激素单药，治疗第10天的总反应率分别为97％、81％，估计1年无失败生存率分别为69％、41％，治疗相关不良事件没有差异[51]。

（2）糖皮质激素联合JAK抑制剂：国内一项前瞻性研究表明，甲泼尼龙（1 mg·kg^−1^·d^−1^）联合JAK2抑制剂芦可替尼（Ruxolitinib）作为一线方案治疗急性GVHD（皮肤53.1％、胃肠道68.8％、肝脏6％）的28天总有效率高达90％以上[52]。目前多中心前瞻随机对照研究正在开展。但前瞻随机双盲研究结果并不支持糖皮质激素联合JAK1抑制剂治疗急性GVHD优于单药糖皮质激素[53]。

3. 其他：糖皮质激素联合mTOR抑制剂：研究发现糖皮质激素联合mTOR抑制剂一线治疗急性GVHD也有一定疗效[54]。

（二）二线治疗

原则上在维持CsA有效浓度基础上加用二线药物，并及时评估疗效，当一种二线药物无效后再换用另一种二线药物。国际上尚无统一的二线药物选择流程，一般遵循各自中心的用药原则。鼓励患者参加临床试验。

1. 芦可替尼：芦可替尼用于糖皮质激素耐药急性GVHD的治疗具有最高级别的循证医学证据[25]。多中心开放随机研究表明，在糖皮质激素耐药急性GVHD，芦可替尼组28 d总反应率明显高于对照组（62％对39％）[55]。推荐用法：成人初始剂量为10 mg/d（分2次口服），3 d后若血液学参数稳定且未发生治疗相关不良反应可调整剂量至20 mg/d。体重≥25 kg的儿童患者，初始剂量为10 mg/d（分2次口服）；体重<25 kg的儿童患者，初始剂量为5 mg/d（分2次口服）。主要不良反应是血液学毒性和增加感染风险（尤其是病毒感染）。

2. 抗白细胞介素2受体抗体（IL-2RA）单抗：来自中国真实世界的研究表明，巴利昔单抗对成人糖皮质激素耐药急性GVHD患者的总有效率达78.7％～86.8％，CR率达60.9％～69.8％；对儿童单倍体移植后糖皮质激素耐药急性GVHD的总有效率达85％，CR率为74％[56]–[57]。巴利昔单抗推荐用法：成人及体重≥35 kg儿童每次20 mg、体重<35 kg儿童每次10 mg，+1、+3、+8 d各给药1次，以后每周1次，使用次数根据病情而定。

3. MTX：MTX是由中国医师最先用于急性GVHD治疗的药物。MTX二线治疗急性GVHD也取得很好疗效，治疗急性GVHD的有效率为94％，治疗DLI后GVHD的有效率为100％，对于皮肤、胃肠道、肝脏GVHD的有效率分别为100％、60％、71％[58]。推荐MTX用法：成人每次10 mg，+1、+3、+8 d各给药1次，以后每周1次，静脉或口服给药。儿童患者酌减。MTX的主要不良反应为血液毒性和口腔溃疡，适用于血象良好且没有口腔溃疡的患者。

（三）其他治疗

间充质干细胞输注可作为急性GVHD二线或三线治疗选择。尽管早期前瞻随机研究未能证实间充质干细胞治疗糖皮质激素耐药的急性GVHD较对照组有优势，但近年来仍有不同研究证实了间充质干细胞的有效性。间充质干细胞对糖皮质激素耐药、二线药物以上多重耐药的急性GVHD总有效率可达57％～73％[59]–[60]。国内多中心前瞻随机对照研究证实，间充质干细胞联合巴利昔单抗治疗糖皮质激素耐药急性GVHD的总有效率明显高于单药巴利昔单抗[61]–[62]。此外，粪菌移植、ATG、维多珠单抗（Vedolizumab）、托珠单抗（Tocilizumab）、英夫利昔单抗（Infliximab）、维布妥昔单抗（Brentuximab vedotin）、抗CCR5单抗等均有进一步研究的潜力。

七、受累器官的局部管理和患者的整体管理

（一）强化受累器官的管理

1. 皮肤受累的急性GVHD：加强局部护理，保持清洁，局部应用皮肤保护剂，减少渗出。

2. 胃肠道受累的急性GVHD：应重视胃肠道休息、减少或停止经口摄入、部分或全部胃肠外营养补充热量，重视水电酸碱平衡，当不能除外肠道感染时给与经验性抗生素进行肠道除菌，不建议积极使用收敛剂对症处理，以免导致诊断评估的延误便血患者加强输血支持。

3. 肝脏受累的急性GVHD：慎用影响肝脏的药物，可应用熊去氧胆酸。

（二）重视患者的整体管理

发生急性GVHD时，除了皮肤或黏膜屏障功能受损，免疫功能也受抑制，易于发生各种严重感染，所以治疗急性GVHD过程中注意感染的监测和预防，如预防疱疹病毒感染、真菌感染，常规监测巨细胞病毒、EB病毒等。

八、总结

急性GVHD的防治在持续进展（尤其是单倍体移植），但急性GVHD仍然是allo-HSCT最常见的合并症和死亡原因之一，规范并优化急性GVHD防治对提高移植的成功率十分重要，期望未来可通过宿主、供者和疾病风险因素的预防方法，实现预防和治疗GVHD方案的个体精准化。本共识将根据相关研究进展和临床实践而不断更新。
